# Cultural Adaptation and Validation of the Japanese Version of the Psychological Safety in High-Fidelity Simulation Scale (PS-HFS-J)

**DOI:** 10.3390/nursrep15070257

**Published:** 2025-07-15

**Authors:** Keisuke Nojima, Makoto Tsukuda, Kosuke Kawamura, Junko Honda, Mie Murozumi

**Affiliations:** 1 Faculty of Nursing, Kyoto Tachibana University, Kyoto 607-8175, Japan; kawamura-k@tachibana-u.ac.jp (K.K.); murozumi@tachibana-u.ac.jp (M.M.); 2 Department of Nursing, Hyogo Medical University, Nishinomiya 663-8501, Japan; ma-tsukuda@hyo-med.ac.jp; 3 Research Institute of Nursing Care for People and Community, University of Hyogo, Akashi 673-8588, Japan; junko_honda@cnas.u-hyogo.ac.jp

**Keywords:** simulation-based education, psychological safety, nursing student, scale validation, nursing education

## Abstract

**Background/Objectives**: Psychological safety is essential for effective learning in high-fidelity simulation (HFS); however, no validated Japanese instrument currently exists to measure psychological safety among nursing students. This study aimed to translate the Psychological Safety in High-Fidelity Simulation (PS-HFS) scale into Japanese (PS-HFS-J) and evaluate its psychometric properties. **Methods**: Following COSMIN guidelines, the PS-HFS was translated through forward and back translation, reviewed by an expert panel, and tested for face validity via pilot testing. The scale’s reliability and validity were subsequently examined in 263 undergraduate nursing students using confirmatory factor analysis (CFA), Cronbach’s alpha, and intraclass correlation coefficients (ICC). **Results**: CFA confirmed a good fit of the original four-factor model (CFI = 0.990, TLI = 0.988, RMSEA = 0.026). The scale demonstrated excellent internal consistency (Cronbach’s α = 0.906 overall) and strong test-retest reliability (ICC range: 0.859–0.914). Content validity indices were also high (I-CVI = 0.80–1.00, S-CVI/Ave = 0.94). **Conclusions**: The PS-HFS-J is a reliable, valid, and culturally adapted instrument for assessing psychological safety in Japanese nursing education. It can support educational research, curriculum development, and faculty training, contributing to safer and more effective simulation-based education. Future studies should examine its applicability across diverse educational levels and clinical contexts.

## 1. Introduction

Simulation-based education (SBE) has become an integral component of nursing curricula worldwide, offering students opportunities to develop clinical reasoning and technical skills in a safe and controlled environment. Numerous studies have demonstrated that SBE enhances knowledge retention [1], critical thinking [2], learner satisfaction [3], and clinical decision-making abilities [4]. Therefore, the aim of this study was to translate the Psychological Safety in High-Fidelity Simulation (PS-HFS) scale into Japanese, hereafter referred to as the PS-HFS-J, and evaluate its psychometric properties—including content validity, construct validity, internal consistency, and test-retest reliability—among undergraduate nursing students in Japan.

High-fidelity simulation (HFS), in particular, has gained attention because it closely replicates real-world clinical environments and allows learners to engage in immersive, realistic scenarios. HFS has been shown to significantly improve nursing students’ knowledge, skills, collaboration, quality of care, and learning motivation, compared to other instructional methods [5,6]. Furthermore, research suggests that the educational effects of simulation may differ depending on the level of fidelity, with HFS providing greater improvements in knowledge, skills, and attitudes [7]. In Japan, simulation-based education is considered effective in improving technical skills, teamwork, clinical judgment, professional identity, self-efficacy and confidence, and strategies for effective learner learning are attracting attention [8,9,10].

Despite these advantages, simulation environments may also induce anxiety [11], fear of evaluation, and hesitation in nursing students, which can hinder learning and performance [12]. One of the key factors influencing effective learning in simulation is psychological safety—the perception that one can ask questions, make mistakes, and express opinions without fear of embarrassment or negative consequences [13]. Although gaps in the perception of psychological safety between students and instructors have been reported, a lack of psychological safety has been associated with reduced learning outcomes [14]. Therefore, promoting psychological safety is essential for creating pedagogically sound simulation experiences. To measure psychological safety among nursing students in HFS environments, Park and Kim (2021) [15] developed the Psychological Safety in High-Fidelity Simulation (PS-HFS) scale. This instrument consists of 14 items across four dimensions: Dealing with Uncertainty, Being Exposed, Being Unsupported, and Interpersonal Risk. It was designed to assess nursing students’ perceptions of psychological safety during high-fidelity simulation exercises. The original Korean version demonstrated strong psychometric properties and provided a valuable tool for assessing the emotional and social dynamics of simulation-based learning.

Although the importance of considering psychological safety in SBE has been recognized in Japan, no validated PS-HFS-J currently exists. Given the cultural and linguistic differences in communication styles, educational norms, and hierarchical relationships between countries, direct application of the original version may not fully capture the experiences of Japanese nursing students. To ensure accurate measurement and support the implementation of safe and effective simulation environments in Japan, culturally appropriate adaptation and validation of the scale are essential. The aim of this study was to translate the PS-HFS scale into Japanese and evaluate its psychometric properties—including content validity, construct validity, internal consistency, and test-retest reliability—among undergraduate nursing students in Japan.

## 2. Materials and Methods

### 2.1. Study Design

This study followed the COSMIN (COnsensus-based Standards for the selection of health Measurement INstruments) guidelines [16] for the translation and validation of patient-reported outcome measures. A methodological study was conducted to cross-culturally adapt the Psychological Safety in High-Fidelity Simulation (PS-HFS) scale into Japanese and to evaluate its psychometric properties in a population of undergraduate nursing students. The process included forward and back translation, expert review, pilot testing for face and content validity, and psychometric testing to examine internal consistency, test-retest reliability, and structural validity. Regarding the nursing students who participated in the study, inclusion criteria were undergraduate nursing students enrolled at participating institutions who provided informed consent. Exclusion criteria were incomplete survey responses.

Informed consent was obtained electronically prior to participation. Participants were provided with a detailed information sheet and were required to confirm consent before proceeding to the survey.

### 2.2. Instrument Development and Translation Process

The original Psychological Safety in High-Fidelity Simulation (PS-HFS) scale, developed by Park and Kim (2021) [15], consists of 14 items across four dimensions: Dealing with Uncertainty, Being Exposed, Being Unsupported, and Interpersonal Risk, designed to assess psychological safety in simulation-based learning environments. Permission to translate and adapt the original scale into Japanese was obtained from the developer. The translation process followed the guidelines for cross-cultural adaptation of patient-reported outcome measures as outlined by the COSMIN initiative. First, two independent bilingual translators (native Japanese speakers with experience in nursing and simulation education) conducted forward translations from English to Japanese. Discrepancies between the two versions were reconciled through consensus discussions with the research team, resulting in a synthesized draft.

Next, the synthesized version was independently back-translated into English by two translators who were blinded to the original scale. The research team compared the back-translations with the original version to ensure conceptual equivalence. Subsequently, a pilot test was conducted with 31 undergraduate nursing students (first-year: 7, second-year: 8, third-year: 7, fourth-year: 9) to assess the clarity and comprehensibility of each item. Items that were rated as unclear by more than 15% of participants were revised. A total of five items were modified during this process. After these revisions, no item was rated as unclear by more than 15% of respondents, confirming the face validity of the translated scale.

An expert panel consisting of two nursing faculty members and one simulation education specialist reviewed the translated items to assess semantic, idiomatic, experiential, and conceptual equivalence. Based on their feedback, further preliminary revisions were made. Items that were rated as “unclear” by more than 15% of the expert panel were revised. Item-level and scale-level content validity indices (I-CVI and S-CVI/Ave) were then calculated. The PS-HFS-J was revised until all items achieved an I-CVI of 0.80 or higher and the overall scale achieved an S-CVI/Ave of 0.90 or higher. The final PS-HFS-J consists of 14 items grouped into four dimensions.

### 2.3. Reliability and Validity

#### 2.3.1. Subsubsection

Participants for the construct validity analysis were recruited via an anonymous online survey distributed to nursing students at a Japanese university. Participants were recruited through social networking services and poster advertisements. The survey was conducted between October 2024 and March 2025. Participation was voluntary and uncompensated.

#### 2.3.2. Test-Retest Reliability

Test-retest reliability was evaluated by administering the PS-HFS scale twice to a subsample of participants. At the end of the initial online survey, participants were invited to voluntarily provide their email address if they were willing to participate in a follow-up survey. Those who consented received a second invitation to complete the same scale three weeks later.

### 2.4. Data Analysis

This study assessed the structural equivalence between the original and PS-HFS-J and examined construct validity using confirmatory factor analysis (CFA). CFA was performed based on the hypothesized four-factor model using maximum likelihood estimation. Multiple fit indices were used to evaluate the model’s goodness of fit, including the root mean square error of approximation (RMSEA), Tucker–Lewis index (TLI), and comparative fit index (CFI). An RMSEA value of less than 0.08 was considered indicative of acceptable model fit, while TLI and CFI values greater than 0.95 were interpreted as evidence of good model fit.

Internal consistency was assessed using Cronbach’s alpha, the most widely used index of internal consistency. A Cronbach’s alpha value of 0.70 or higher was considered acceptable. Items with more than 50% missing responses in any dimension were excluded from the dataset prior to analysis. Test-retest reliability was assessed using a two-way random effects intraclass correlation coefficient (ICC) model. ICC values were interpreted as follows: <0.50 = poor, 0.50–0.75 = moderate, 0.75–0.90 = good, and ≥0.90 = excellent reliability. All statistical analyses were performed using IBM SPSS Statistics version 30 and IBM AMOS version 30 (IBM Corp., Armonk, NY, USA).

### 2.5. Ethical Considerations

The study protocol was approved by the Institutional Review Board of the institution the authors belong to (Approval No. 24-15) on 23 May 2024 and conducted in accordance with the Declaration of Helsinki and ethical guidelines for human research. Participation in the study was voluntary and anonymous. No identifying information such as names, address, or IP addresses was collected. All participants were informed of the purpose and procedures of the study and were required to read a consent form before beginning the questionnaire. Returning the completed online questionnaire was considered as implied consent to participate in the study. For participants who agreed to take part in the test-retest reliability assessment, email addresses were voluntarily provided at the end of the first survey and were stored securely and separately from the response data to ensure confidentiality.

## 3. Results

### 3.1. Participants

A total of 278 responses were collected from undergraduate nursing students. After excluding 15 responses due to incomplete or missing data, 263 valid responses were retained for the final analysis to evaluate the reliability and validity of the PS-HFS-J. Among the participants, 44 were first-year students (16.7%), 62 were second-year students (23.6%), 88 were third-year students (33.5%), and 69 were fourth-year students (26.2%).

### 3.2. Face and Content Validity

A pilot test was conducted with 31 nursing students (first-year: 7, second-year: 8, third-year: 7, fourth-year: 9), along with input from a panel of expert reviewers. During this process, three items were identified as unclear by more than 15% of participants:

Item 3 “I feel like I am thrown into the class unprepared.”;

Item 12 “My peers will not criticize me for my mistakes.”;

Item 13 “I do not feel ashamed of showing my peers my mistakes.”

Based on discussions within the research team and feedback from the expert panel, a total of five items—including the three mentioned above—were revised to improve clarity and readability, while preserving the original intent of the items. The final PS-HFS-J consisted of 14 items across four conceptual domains. A follow-up evaluation confirmed that no items were rated as unclear by more than 15% of participants. These findings support the face validity of the PS-HFS-J, which was subsequently finalized and subjected to psychometric testing. The item-level content validity index (I-CVI) for all items ranged from 0.80 to 1.00, and the average scale-level content validity index (S-CVI/Ave) was 0.94, indicating strong content validity.

### 3.3. Construct Validity

Construct validity was evaluated using confirmatory factor analysis (CFA) based on the hypothesized four-factor model. The model demonstrated a good fit to the data: χ^2^ (71) = 83.784, *p* = 0.142; GFI = 0.958; AGFI = 0.937; CFI = 0.990; TLI = 0.988; and RMSEA = 0.026 (90% CI = [0.000–0.060]). These results support the structural validity of the PS-HFS-J. The confirmatory factor analysis indices are listed in Table 1. Table 2 shows the standardized factor loadings for each item in the PS-HFS-J.

### 3.4. Reliability

The reliability of the scale was assessed using the test-retest method. A total of 52 participants completed the test at both time points. Of these, 14 were first-year students (26.9%), 10 were second-year students (19.2%), 18 were third-year students (34.6%), and 10 were fourth-year students (19.2%).

#### 3.4.1. Internal Consistency

Cronbach’s alpha coefficients were calculated to evaluate the internal consistency of each factor of the PS-HFS-J. The overall Cronbach’s alpha coefficient for the entire scale was 0.906, indicating excellent internal consistency. Each of the four subscales also demonstrated high reliability, with alpha coefficients above the commonly accepted threshold of 0.70. (Table 3).

#### 3.4.2. Intraclass Correlation Coefficients for the Test-Retest Method

The ICCs for each dimension ranged from 0.859 to 0.914, and the lower bound of the 95% confidence interval was 0.790, suggesting that the scale has at least moderate stability over time (Table 4).

## 4. Discussion

### 4.1. Construct Validity and Reliability

Construct validity was examined using confirmatory factor analysis (CFA). The results demonstrated good model fit, with values of CFI = 0.990, TLI = 0.988, and RMSEA = 0.026 (90% CI = [0.000–0.060]). According to Hair et al. (2019) [17], values of CFI and TLI above 0.95 and an RMSEA below 0.06 indicate a good fit, while values above 0.90 are considered acceptable. Similarly, Schreiber et al. (2006) [18] have suggested that in the fields of education and behavioral sciences, CFI and TLI values greater than 0.95 and RMSEA values less than 0.06 are indicative of a well-fitting model.

These findings align with those reported in the original version the PS-HFS developed by Park and Kim (2021) [15], supporting the scale’s hypothesized four-factor structure. They are also consistent with prior validation studies that emphasize the multidimensional nature of psychological safety in simulation-based learning environments. Based on these widely accepted standards, the CFA results obtained in this study provide strong evidence for the structural validity of the PS-HFS-J.

The reliability of the PS-HFS-J was examined through both internal consistency and test–retest stability. Cronbach’s alpha coefficients for the overall scale and each of the four subscales exceeded the commonly accepted threshold of 0.80, indicating high internal consistency. Test–retest reliability was assessed using intraclass correlation coefficients (ICCs), calculated based on a two-way random effects model. The ICCs for the four factors ranged from 0.859 to 0.914, with the lower bound of the 95% confidence intervals exceeding 0.790. According to established criteria by Koo and Li (2016) [19], ICC values below 0.5 are considered poor, between 0.5 and 0.75 moderate, 0.75 to 0.9 good, and values above 0.9 excellent. Based on these benchmarks, the PS-HFS-J demonstrated good to excellent temporal stability across all subscales. These findings indicate that the PS-HFS-J reliably captures perceptions of psychological safety in high-fidelity simulation over time, making it a suitable instrument for use in both cross-sectional and longitudinal evaluations within nursing education contexts.

### 4.2. Educational Implications

The validated PS-HFS-J offers educators a practical tool to assess nursing students’ perceived psychological safety during simulation. Although the current study did not directly test educational interventions, prior research suggests that psychological safety can be promoted through structured prebriefings, supportive facilitator behaviors, and clear communication strategies [20,21]. Identifying areas of vulnerability through PS-HFS-J could inform simulation design and faculty development efforts aimed at enhancing the emotional and interpersonal quality of the learning environment. Furthermore, Silva et al. (2022) [22] demonstrated that simulation-based education significantly reduces anxiety (d = −0.33, *p* = 0.034) and increases self-confidence (d = 0.71, *p* < 0.001), both of which contribute positively to learning outcomes. These findings underscore the relevance of measuring and supporting psychological safety as part of comprehensive simulation pedagogy.

Beyond undergraduate nursing education, the PS-HFS-J has potential applicability in interprofessional education, continuing professional development, and clinical team simulations to support psychological safety among diverse healthcare providers. Although this study collected data specifically from undergraduate nursing students, future research should explore and validate the scale’s use in these broader contexts to ensure its appropriateness and effectiveness across settings.

### 4.3. Cultural Considerations

While psychological safety is a relevant construct across educational settings, its perception and expression may vary by cultural context. In collectivist cultures like Japan, social harmony, avoidance of shame, and deference to hierarchy are strongly emphasized [23,24]. These cultural norms may influence learners’ reluctance to speak up, ask questions, or acknowledge mistakes in front of peers or instructors.

Ito et al. (2022) [25] highlighted that psychological safety in healthcare settings in East Asia is shaped by status differentials, fear of criticism, and interpersonal risk. Such factors may lead students to underreport distress or avoid behaviors that appear confrontational, even in psychologically safe environments. Cultural nuances may have influenced how participants interpreted certain items, particularly those related to peer judgment and personal exposure (e.g., Items 12 and 13). Moreover, Banks (2016) [24] describes three factors behind students’ choice to remain silent in order to maintain relationships with teachers and classmates: “group communication system,” “respect for teachers and peers,” and “high expectations for speaking up.”

Despite these cultural nuances, the successful replication of the original four-factor structure in the PS-HFS-J suggests that core elements of psychological safety—such as support, trust, and freedom from interpersonal risk—are relevant across cultures. Nevertheless, simulation educators should adopt culturally responsive strategies, such as role modeling vulnerability, explicitly inviting questions, and normalizing mistakes during prebriefings and debriefings [25,26].

### 4.4. Limitations and Future Directions

This study has several limitations. First, participants were recruited from a limited number of institutions, which may restrict the generalizability of findings. Second, data were based on self-reported responses, potentially subject to social desirability or recall bias. Third, the scale was tested exclusively with undergraduate nursing students; its applicability to other learner populations (e.g., clinical nurses, interprofessional teams) remains unknown. Future research should evaluate the PS-HFS-J across diverse educational levels and professional contexts. Longitudinal studies could examine how psychological safety evolves over time or in response to specific simulation interventions. Mixed-methods studies may also provide deeper insights into the emotional and interpersonal dynamics underlying learners’ experiences in HFS. Finally, implementation studies are needed to determine the feasibility and utility of the PS-HFS-J as part of routine simulation practice and curriculum evaluation.

## 5. Conclusions

This study successfully translated and validated the Japanese version of the Psychological Safety in High-Fidelity Simulation (PS-HFS-J) scale for use among undergraduate nursing students. The scale demonstrated strong psychometric properties, including excellent internal consistency, good to excellent test-retest reliability, and a well-fitting four-factor structure that mirrors the original version. The process adhered to COSMIN guidelines to ensure linguistic and cultural appropriateness.

The PS-HFS-J provides a reliable and culturally adapted instrument for assessing psychological safety in high-fidelity simulation environments within Japanese nursing education. Its use can support educational research, curriculum design, and faculty development by enabling educators to identify emotional and interpersonal factors that influence student learning. As simulation-based education continues to expand, tools such as the PS-HFS-J will play a critical role in creating psychologically safe and learner-centered environments that promote clinical competence and reflective practice.

Future studies should explore the scale’s applicability in other learner populations, clinical contexts, and educational levels, as well as its integration into simulation pedagogy and faculty training. Broader use of the PS-HFS-J will contribute to the advancement of evidence-based simulation practices in Japan and beyond.

## Figures and Tables

**Table 1:** Confirmatory factor analysis of PS-HFS-J.

Fit Indices	Result	
*Χ*^2^ (71)	83.784	
*p*	0.142	
GFI	0.958	
AGFI	0.937	
CFI	0.99	
TLI	0.988	
RMSEA	0.026	90% CI [0.000–0.060]

Abbreviations: GFI: Goodness-of-Fit Index, AGFI: Adjusted Goodness-of-Fit Index, CFI: Comparative Fit Index, TLI: Tucker-Lewis Index, RMSEA: root-mean-square error of approximation.

**Table 2:** Standardized factor loadings for each item in the PS-HFS-J.

Factor	Item No.	Standardized Loading
Dealing with Uncertainty	1	0.72
	2	0.74
	3	0.73
	4	0.72
Being Exposed	5	0.76
	6	0.77
	7	0.95
	8	0.76
Being Unsupported	9	0.80
	10	0.78
	11	0.79
Interpersonal Risk	12	0.74
	13	0.75
	14	0.76

**Table 3:** Cronbach’s alpha in each factor.

Factor	Cronbach’s Alpha	95% CI	*p*-Value
Dealing with Uncertainty	0.816	0.691–0.880	<0.001
Being Exposed	0.811	0.682–0.873	<0.001
Being Unsupported	0.778	0.601–0.829	<0.001
Interpersonal Risk	0.821	0.689–0.884	<0.001

Abbreviation: CI: Confidence Interval.

**Table 4:** Intraclass correlation of test-retest method.

Total and Subscale Scores	ICC	95% CI	*p*-Value
Dealing with Uncertainty	0.859	0.790–0.918	<0.001
Being Exposed	0.888	0.811–0.937	<0.001
Being Unsupported	0.914	0.845–0.953	<0.001
Interpersonal Risk	0.886	0.801–0.936	<0.001
Total score	0.889	0.804–0.939	<0.001

Abbreviations: ICC: Intraclass Correlation Coefficient; CI: Confidence Interval.

## Data Availability

The data presented in this study are available on request from the corresponding author.

## Public Involvement Statement

No public involvement in any aspect of this research.

## Guidelines and Standards Statement

This manuscript was drafted against the COSMIN (COnsensus-based Standards for the selection of health Measurement INstruments) guideline for studies on measurement properties [16].

## Abbreviations

The following abbreviations are used in this manuscript:

AGFI	Adjusted Goodness-of-Fit Index
CFA	Confirmatory factor analysis
COSMIN	COnsensus-based Standards for the selection of health Measurement INstruments
GFI	Goodness-of-Fit Index
HFS	High-Fidelity Simulation
ICC	Intraclass Correlation Coefficients
I-CVI	Item-Level Content Validity Indices
PS-HFS	Psychological Safety in High-Fidelity Simulation
RMSEA	Root-Mean-Square Error of Approximation
S-CVI	Scale-Level Content Validity Indices
TLI	Tucker-Lewis Index
